# Evaluation of Binding Effects in Wood Flour Board Containing Ligno-Cellulose Nanofibers

**DOI:** 10.3390/ma7096853

**Published:** 2014-09-22

**Authors:** Yoichi Kojima, Akiko Isa, Hikaru Kobori, Shigehiko Suzuki, Hirokazu Ito, Rie Makise, Masaki Okamoto

**Affiliations:** 1 Faculty of Agriculture, Shizuoka University, Shizuoka 422-8529, Japan; E-Mails: aaisa@ipc.shizuoka.ac.jp (A.I.); kobori.hikaru@shizuoka.ac.jp (H.K.); afssuzu@ipc.shizuoka.ac.jp (S.S.); 2 Toclas Corporation, Hamamatsu 432-8001, Japan; E-Mails: hirokazu_ito@toclas.co.jp (H.I.); rie_makise@toclas.co.jp (R.M.); masaki_okamoto@toclas.co.jp (M.O.)

**Keywords:** wood-based materials, wood flour, ligno-cellulose nanofiber, pulverize

## Abstract

Wood-based materials are used extensively in residual construction worldwide. Most of the adhesives used in wood-based materials are derived from fossil resources, and some are not environmentally friendly. This study explores nanofiber technology as an alternative to such adhesives. Previous studies have shown that the three-dimensional binding effects of cellulose nanofiber (CNF), when mixed with wood flour, can significantly improve the physical and mechanical properties of wood flour board. In this study, ligno-cellulose nanofibers (LCNF) were fabricated by wet disk milling of wood flour. Composite boards of wood flour and LCNF were produced to investigate the binding effect(s) of LCNF. The fabrication of LCNF by disk milling was simple and effective, and its incorporation into wood flour board significantly enhanced the physical and mechanical properties of the board.

## 1. Introduction

Wood-based materials are used worldwide in residential construction, especially in Japan. These materials can be made from virgin or recycled wood, and many are fabricated with various adhesives. Most of the currently available wood adhesives, such as formaldehyde-based resins, vinyl acetate resins, and isocyanate-based resins, are composed of various materials derived from fossil resources. It is likely that many modern wood adhesives will be restricted in the future due to depletion of fossil resource reserves. Moreover, several modern adhesives are not environmentally friendly. Finding replacements for such chemical adhesives poses major challenges. The global effort toward sustainability demands the development of a novel, natural adhesive that does not depend on fossil resources and synthetic chemicals. Many studies have focused on developing natural material-based adhesives using bio-resources [1]. For example, a natural adhesive, composed of citric [2,3,4,5] and lactic acids [6,7] for use in wood-based materials has been reported.

Nanotechnology is developing rapidly in multiple fields. In general, the term nanofiber refers to a nano-sized fiber with a diameter between 1 and 100 nm and an aspect ratio of more than 100. In addition, a fiber that has a surface and inner structure controlled at the nanoscale level is called a nanostructured fiber [8]. This is true even for fibers having diameters exceeding 100 nm. Extensive research has been conducted on the development and application of cellulose nanofiber (CNF), which boasts better physical and mechanical properties than most other natural fibers [9]. The development of new materials incorporating CNF is an active goal in many fields [10,11,12,13,14]. However, to the best of our knowledge, the use of CNF technology in wood-based materials has not been reported. However, some researches about the relationship between fiber shape and mechanical properties for MDF were reported [15,16,17], there was no discussion about nano-order fiber.

In our previous work [18], we investigated the effects of adding CNF to wood flour. The resulting properties of the CNF/wood flour boards were evaluated with a focus on the binding effects of CNF. We observed that wet ball-milling of commercial cellulose powder led to the formation of nanostructured fibers with nano-sized surface fibrils. Moreover, the physical and mechanical properties of the wood flour boards were significantly enhanced by the addition of CNF due to three-dimensional binding between the CNF and wood flour.

This report describes the fabrication of ligno-cellulose nanofibers (LCNF) from wood flour. In this context, CNF refers to nanofibers made from cellulose alone. A wet-pulverising method was developed to make LCNF using a disk mill, and the morphology of the resulting fibers was studied. The binding effects of LCNF with wood flour were investigated as a function of LCNF content and particle size.

## 2. Materials and Methods

### 2.1. Materials

Recycled wood flour consisting mainly of particleboard was obtained from Toclas Corporation (Shizuoka, Japan). The median size of the wood flour fibers was about 130 μm. Note that wood flour was used both as a base material for wood flour board and as a raw material for LCNF.

### 2.2. Pulverisation of Wood Flour to Make LCNF

Wood flour was mixed with water at a weight ratio of 5:95, and the resulting slurry was kept for two days. The slurry was then pulverized in a multistep process in a disk mill (MKZA10-15J; Masuko Sangyo Co., Ltd., Saitama, Japan) at 2000 rpm. The degree of pulverization was controlled by the disk clearance and the number of repeated passes through the disk, *i.e.*, pass number. The pulverizing conditions are shown in Table 1. The pass number was set from 0 to 8. The initial disk clearance was set to 250 μm and was decreased every two passes. The final 7th and 8th passes were with a disk clearance of 160 μm. Nine different LCNF slurries (W0 through W8) were evaluated. A slurry made from untreated (non-pulverized) WF was used as a control. The particle size of the LCNF after pulverization was measured using a laser diffraction particle size distribution analyzer (Partica LA-950; Horiba, Ltd., Kyoto, Japan). To prevent flocculation, the LCNF slurry was replaced with alcohol. The surface morphology of the resulting LCNF powder was observed with a scanning electron microscope (SEM) (TM1000; Hitachi, Tokyo, Japan).

**Table 1:** Pulverizing conditions and disk mill settings.

Samples	Disk clearance (μm)														
	1pass		2pass		3pass		4pass		5pass		6pass		7pass		8pass
W0	-		-		-		-		-		-		-		-
W1	250		-		-		-		-		-		-		-
W2	250	►	250		-		-		-		-		-		-
W3	250	►	250	►	200		-		-		-		-		-
W4	250	►	250	►	200	►	200		-		-		-		-
W5	250	►	250	►	200	►	200	►	180		-		-		-
W6	250	►	250	►	200	►	200	►	180	►	180		-		-
W7	250	►	250	►	200	►	200	►	180	►	180	►	160		-
W8	250	►	250	►	200	►	200	►	180	►	180	►	160	►	160

### 2.3. Fabrication of Wood Flour Board

Wood flour boards were made from mixtures of wood flour and three of the LCNF slurries (W4, W6, and W8) noted above. This allowed evaluation of the physical and mechanical properties of the board composites as a function of LCNF processing. A single composition of 85 wt% wood flour and 15 wt% LCNF was examined. As another experiment, the physical and mechanical properties of the wood flour board were also evaluated as a function of the relative amounts of LCNF and wood flour. The LCNF used in these experiments was W8. Three wood flour:LCNF compositions were evaluated: 85:15, 90:10, and 95:5.

Wood flour was mixed with the LCNF slurry in a polyethylene bag by hand. The mixture was covered over in a metal flame, and a mat (15 × 15 cm) was made. Wire screens were placed on the upper and lower surfaces of the formed mat to accelerate water transfer during subsequent pressing. The hand-formed mats were pressed for 10 min at 120 °C and 1.0 MPa using a hot press (Tabletop Test Press SA-302; Tester Sangyo Co., Ltd., Tokyo, Japan). The resulting wood flour/LCNF boards measured 15 × 15 × 0.3 cm with a target density of 1.00 g/cm^3^.

For all experiments, two boards were produced for each condition evaluated. All of the boards were conditioned at 20 °C and 65% relative humidity for at least two weeks before testing. No adhesives or other additives were used.

### 2.4. Physical Testing

After conditioning, six pieces measuring 12 cm × 2 cm were cut from each wood flour board for three-point bending tests using the following conditions: span 10 cm, loading speed 1 cm/min. The modulus of rupture (MOR) and modulus of elasticity (MOE) were calculated.

Water absorption was determined by measuring the change in weight and thickness of the test pieces before and after soaking in water at 20 °C for 24 h. Unstressed parts of bending test specimens were used for evaluating water absorption.

## 3. Results and Discussion

### 3.1. Evaluation of LCNF Structure

**Figure 1:**
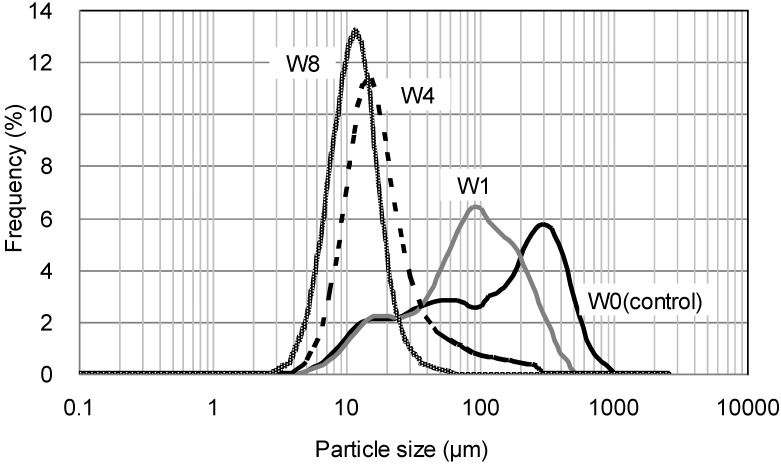
Particle size distributions are shown for W0, W1, W4, and W8.

Table 2 shows the median particle size of LCNF after disk milling. Smaller particle sizes were obtained with increased pass number. The particle size of the W8 LCNF was less than one tenth that of W0 (control). Figure 1 shows the particle size distributions of W0 (control), W1, W4, and W8. The variation in the particle size of the control was relatively large. Smaller variations in particle size were obtained with increased pass number. Therefore, it is possible to control the size uniformity of wood flour particles by adjusting the settings of the disk mill and/or the pass number.

**Table 2:** Median wood flour particle sizes after disk mill pulverization.

Median median particle size of LCNF after disk milling									
**Samples**	W0	W1	W2	W3	W4	W5	W6	W7	W8
**Median particle size (μm)**	126.9	79.3	54.0	19.7	15.2	12.0	12.0	11.1	10.5

**Figure 2:**
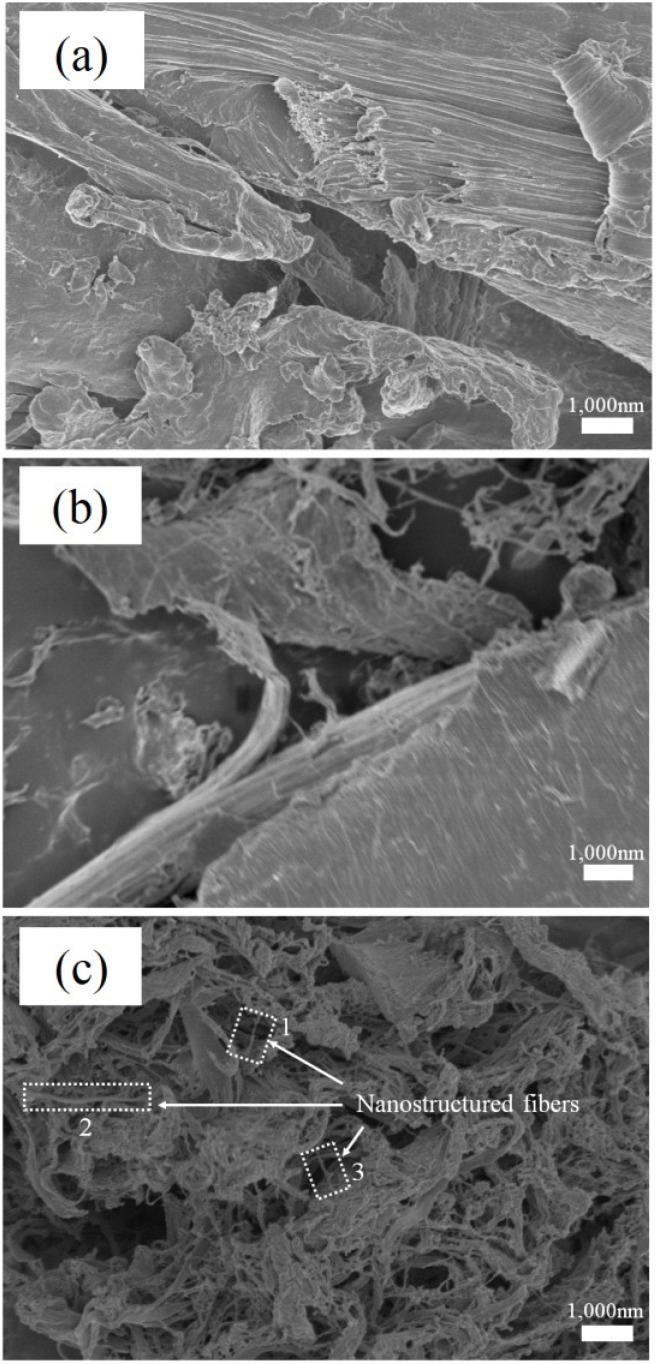
SEM micrographs show the surface morphology of (**a**) untreated WF; (**b**) LCNF (W4); and (**c**) LCNF (W8). Representative nanostructured fibers were enclosed by dotted frame. The diameter of each nanostructured fibers are approximately (1) 66.4 nm, (2) 97.9 nm, and (3) 87.4 nm, respectively.

The SEM micrographs in Figure 2 show the surface morphology of the wood flour samples. The surface of the control wood flour (W0) was smooth, while rougher surfaces and finer pulverization were observed for the disk-milled wood flour. As shown in Figure 2c, nanostructured fibers with nanoscale surface fibrils were formed on the surface of W8 after disk milling. We have confirmed that the size of nanofibrils for the LCNF made by disk-milling is same as of nanofibrils for CNF made by ball-milling [18].

### 3.2. The Binding Effects of LCNF in Wood Flour Boards

**Figure 3:**
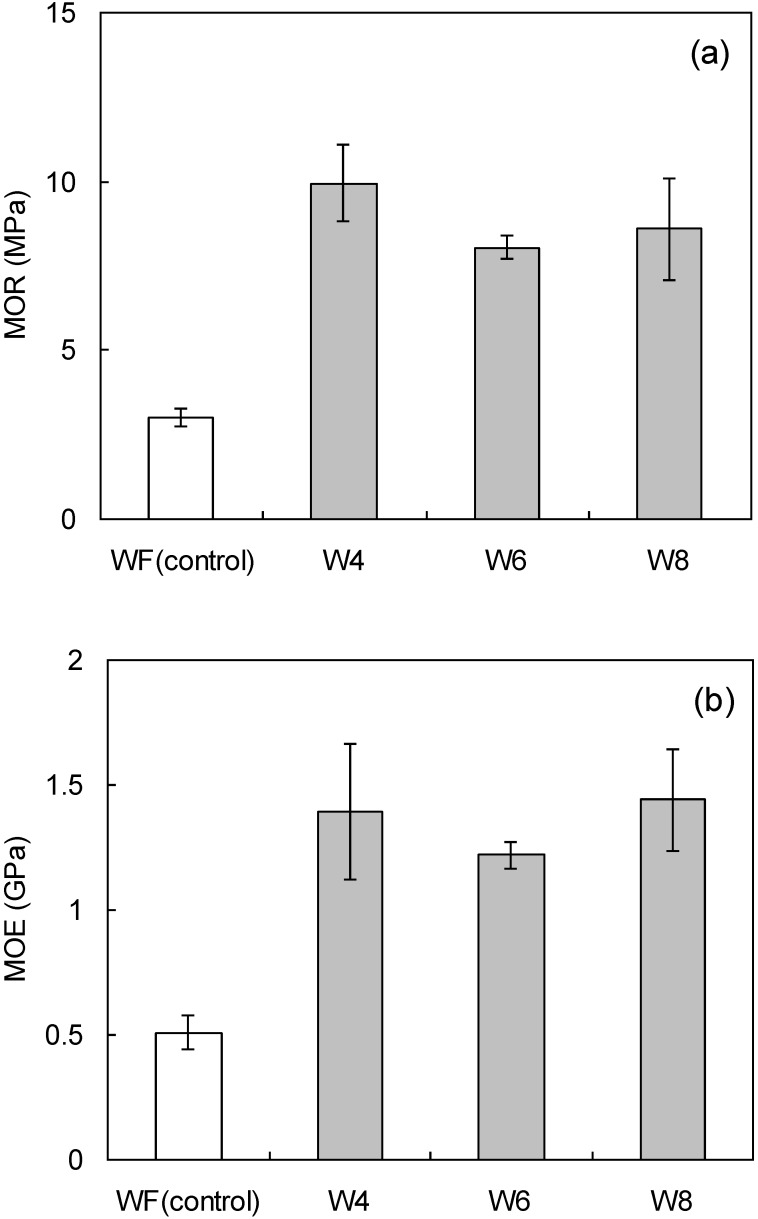
The bending properties, (**a**) MOR and (**b**) MOE, are given for 85:15 wood flour:LCNF boards as a function of pulverization setting. The vertical bars indicate a single standard deviation. WF: wood flour.

Wood flour boards were fabricated from 85:15 wood flour:LCNF to evaluate the binding effects of LCNF (Table 3). The measured board densities were 0.96–0.97 g/cm^3^, very close to the target density of 1.00 g/cm^3^. Figure 3a,b show the bending properties of the wood flour/LCNF boards. According to JIS standard [19], the minimum requirement of MOR for MDF is 20 MPa, and the maximum value of the board in this study is about 10 MPa because of no resin. The MOR and MOE of the composite boards were more than twice those of boards made with only wood flour. This indicates binding between LCNF and wood flour particles. Based on our previous research [18], bending property of wood board with adding CNF of ball milling increased twice as higher than that of wood board without adding CNF. In the case of this research, the bending property of wood board with the adding to LCNF of disk milling also increased twice higher than that of wood board without adding LCNF (see Figure 3. Thus, the LCNF made by disk-milling and CNF made by ball-milling may have same binding ability.

Board bending properties did not differ significantly as a function of LCNF particle size. In our previous paper [18], the bending properties of wood flour board tended to increase with smaller CNF particle sizes. The difference between the effects of CNF and LCNF are due to the size differences between particles of base wood flour and LCNF. As the binder becomes larger, the three-dimensional binding effect becomes smaller.

**Figure 4:**
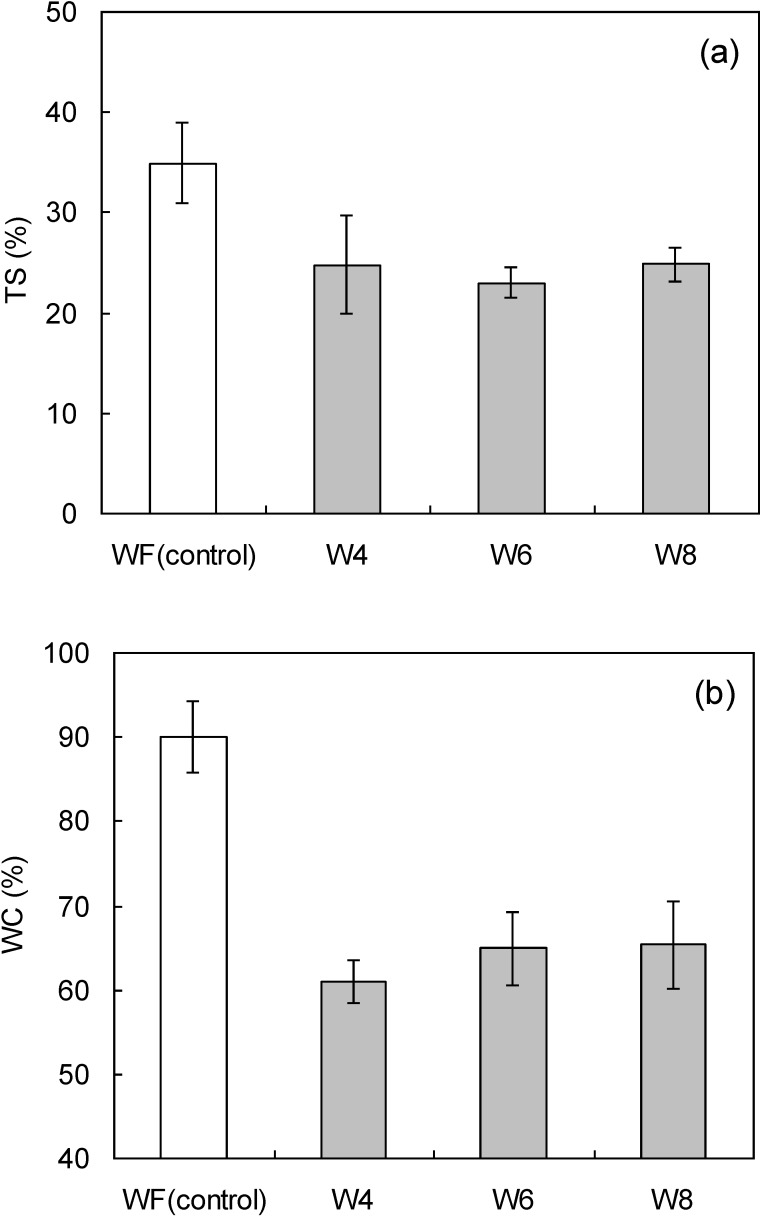
(**a**) Thickness swelling (TS) and (**b**) weight change (WC) of wood flour/LCNF composites are given following water absorption as a function of pulverization setting. The vertical bars indicate a single standard deviation. WF: wood flour.

Figure 4 shows the degrees of thickness swelling (TS) and weight change (WC) in the wood flour/LCNF composites with water absorption. The TS and WC values for boards containing LCNF were lower than those for boards fabricated with wood flour only. The board densities were almost the same as those shown in Table 3, indicating that the void ratios in the test samples were nearly identical. Nano-sized fibrils formed on the fiber surfaces during the mixing of LCNF with wood flour resulted in close binding of the two components. As a result, water was not as easily able to enter the composite samples. Thus, incorporation of LCNF improved water resistance. There was no significant difference in water absorption as a function of LCNF particle size.

**Figure 5:**
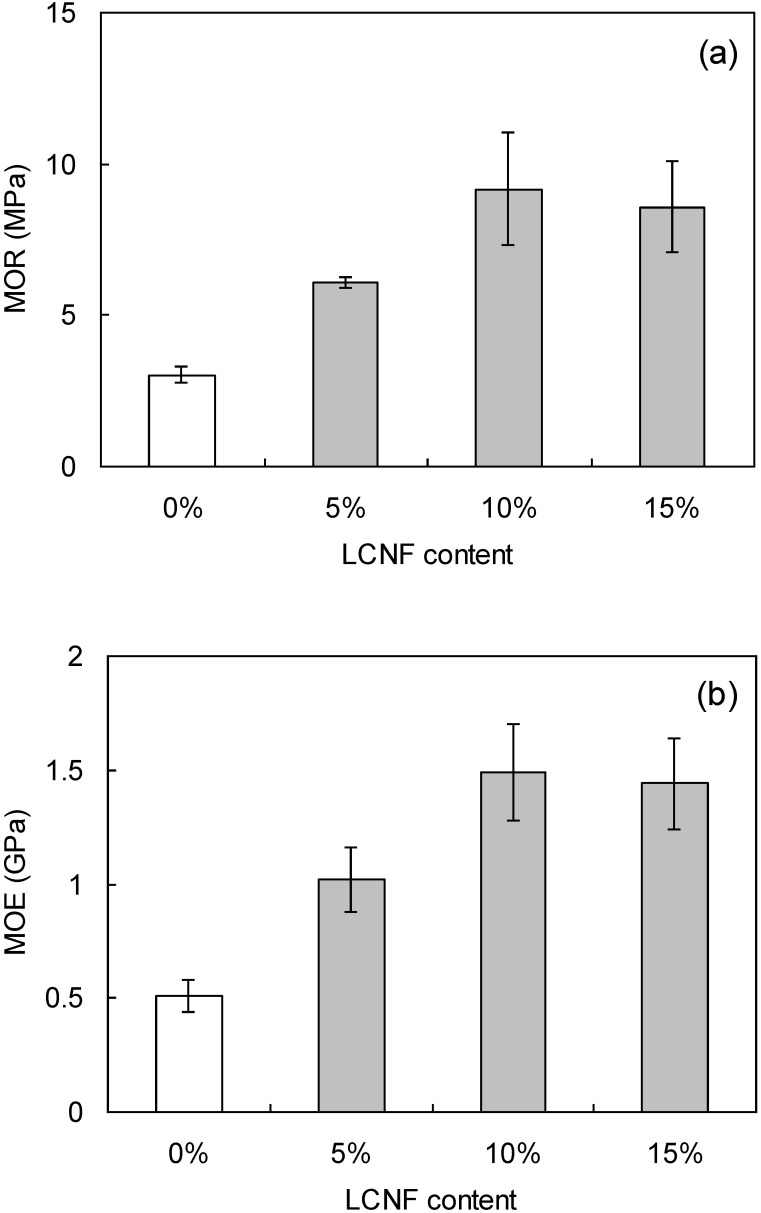
The bending properties, (**a**) modulus of rupture (MOR) and (**b**) and modulus of elasticity (MOE) are given for W8 composites as a function of LCNF content. The vertical bars indicate a single standard deviation.

**Figure 6:**
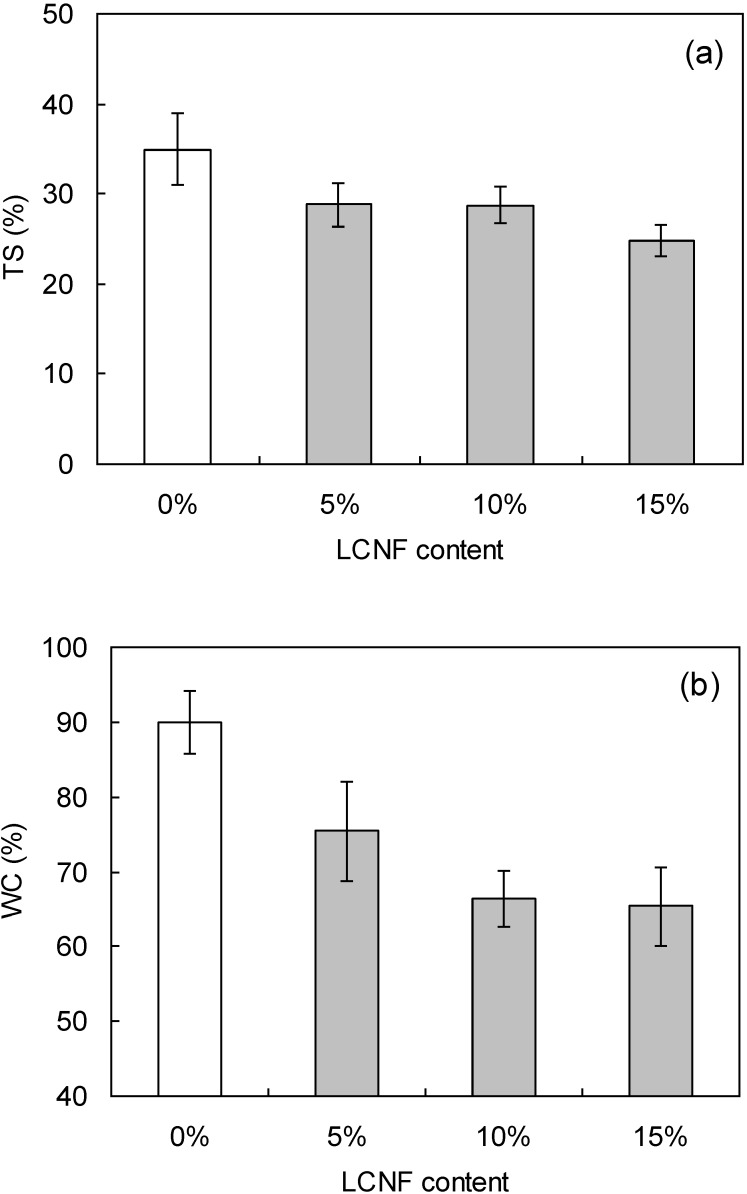
(**a**) TS and (**b**) WC of wood flour/LCNF composites are given following water absorption as a function of LCNF content. The vertical bars indicate a single standard deviation.

The physical and mechanical properties of wood flour boards were also studied as a function of LCNF/wood flour ratio. Note that only the W8 material was used for this study. Table 4 shows the measured density of the manufactured boards. As above, the measured board densities were 0.97–0.99 g/cm^3^, very close to the target density of 1.00 g/cm^3^. Figure 5 shows the bending properties (MOR, MOE) of the boards as a function of LCNF content. The highest values were obtained with boards composed of 90:10 wood flour:LCNF. Both moduli were almost same value above 10%. This was attributed to flocculation of the LCNF, which likely became more important as pulverization progressed. Similar results were obtained for wood flour boards mixed with CNF in our previous report [18]. Figure 6 shows the TS and WC of the composites due to water absorption. Both TS and WC decreased with increasing LCNF content due to close binding of the LCNF to wood flour.

**Table 3:** Board densities are given as a function of pulverization setting.

Samples	Density (g/cm^3^)	Standard deviation
WF (control)	0.97	0.02
W4	0.96	0.02
W6	0.97	0.01
W8	0.97	0.03

WF: wood flour.

**Table 4:** Board densities are given as a function of lingo-cellulose nanofibers (LCNF) content.

Samples	Density (g/cm^3^)	Standard Deviation
WF (control)	0.97	0.02
WF:LCNF = 95:5	0.97	0.03
WF:LCNF = 90:10	0.99	0.02
WF:LCNF = 85:15	0.97	0.03

WF: wood flour.

## 4. Conclusions

Wet disk milling of recycled wood flour resulted in the formation of nano-structured fibers with nano-sized surface fibrils. These fibers are referred to as LCNFs. This study evaluated the binding effects of LCNF in wood flour boards. The uniformity of the processed wood flour was controlled by adjusting the disk mill settings and number of passes during pulverization. The physical and mechanical properties of the resulting wood flour boards were significantly improved by the inclusion of LCNF due to close binding between LCNF and wood flour particles.
